# Distribution of Non-Fermenting Gram-Negative Bacilli and Carbapenem Resistance Profiles in Intensive Care Units of a Foundation University Hospital: A 2023–2025 Retrospective Analysis

**DOI:** 10.3390/medicina62091728

**Published:** 2026-09-08

**Authors:** Hacer Ozturk Akin, Ozge Unlu

**Affiliations:** 1 Department of Medical Laboratory Techniques, Vocational School of Health Services, Istanbul Atlas University, Istanbul 34408, Türkiye; 2 Department of Medical Microbiology, Faculty of Medicine, Istanbul Atlas University, Istanbul 34408, Türkiye

**Keywords:** *Acinetobacter baumannii*, *Pseudomonas aeruginosa*, carbapenem resistance, non-fermenting Gram-negative bacteria, intensive care unit, antimicrobial stewardship, antimicrobial susceptibility, resistance surveillance, Turkey

## Abstract

*Background and Objectives*: Non-fermenting Gram-negative bacilli (NFGNB) are frequently recovered from respiratory specimens in intensive care units (ICUs) and are increasingly carbapenem-resistant. We described their distribution and resistance profiles in adult ICUs over three years. *Materials and Methods*: Respiratory specimens submitted from adult ICUs of a foundation university hospital in Istanbul between 6 January 2023 and 27 December 2025 were analysed retrospectively using laboratory information system data. Identification and susceptibility testing used the BD Phoenix M50 system with year-specific EUCAST breakpoints (v13.0–v15.0); colistin MICs were determined by broth microdilution. The number of patients contributing more than one isolate was 268. Accordingly, the primary trend analysis was performed using generalised estimating equations (GEE) accounting for within-patient clustering. Chi-square and first-isolate analyses were used as secondary and sensitivity analyses. *Results*: A total of 1407 isolates were recovered from 654 of 1194 patients with a respiratory culture. *Acinetobacter* spp. predominated (56.9% of isolates; 54.3% of patients), followed by *Pseudomonas* spp. (29.4%) and *Stenotrophomonas maltophilia* (11.1%). Carbapenem resistance in *Acinetobacter* spp. was 96.7% (95% CI 95.3–97.8) for imipenem and 96.6% (95.1–97.7) for meropenem, without temporal trend. In *P. aeruginosa*, meropenem resistance rose from 44.7% to 71.4% (GEE odds ratio per year 1.45, 95% CI 1.09–1.92, *p* = 0.011), whereas imipenem resistance did not change significantly. Among the agents tested, colistin was found to be the most effective (22.4% resistance in *Acinetobacter* spp. and 13.1% in *P. aeruginosa*). Ceftazidime–avibactam resistance in *P. aeruginosa* was 24.3%. Of the *S. maltophilia* isolates, 85.8% were categorised as susceptible-increased exposure to trimethoprim–sulfamethoxazole. *Conclusions*: Carbapenem resistance is high and stable in *Acinetobacter* spp. but rising for meropenem in *P. aeruginosa*. These data do not distinguish colonisation from infection and cannot be linked to clinical outcomes. They should therefore be used to inform local empirical-treatment policy alongside clinical assessment, rather than as evidence of treatment failure.

## 1. Introduction

Intensive care units (ICUs) concentrate the most vulnerable hospitalised patients and the heaviest antimicrobial pressure. Respiratory tract infections are among the most frequent infections in this setting and carry substantial morbidity and mortality [1,2,3,4]. Invasive airway devices and the severity of underlying illness leave these patients particularly exposed to opportunistic organisms [1]. Among the Gram-negative organisms recovered from ICU respiratory specimens, non-fermenting Gram-negative bacilli (NFGNB) form the subgroup with the fewest remaining treatment options. *Pseudomonas aeruginosa*, the *Acinetobacter baumannii/calcoaceticus* complex and *Stenotrophomonas maltophilia* are the species most often implicated [3,5,6], and they play a central role in ventilator-associated pneumonia [4,5].

NFGNB do not ferment glucose, survive for long periods on hospital surfaces and in moist environments, and tolerate many disinfectants. Beyond the three principal species, *Burkholderia cepacia* complex, *Achromobacter* spp., *Sphingomonas paucimobilis*, *Chryseobacterium* spp. and *Elizabethkingia* spp. are recovered less frequently but are also characterised by extensive intrinsic resistance [2,3,7,8].

Carbapenem resistance arises through different mechanisms in these organisms. This also bears on how phenotypic surveillance data should be interpreted. In the *A. baumannii/calcoaceticus* complex, resistance is driven predominantly by acquired OXA-type carbapenem-hydrolysing class D β-lactamases—particularly OXA-23, OXA-24/40 and OXA-58—frequently mobilised by upstream IS*Aba1* insertion sequences and less commonly by metallo-β-lactamases such as NDM. These enzymatic mechanisms act together with RND-family efflux systems (AdeABC, AdeIJK, AdeFGH) and with loss or alteration of the CarO and Omp33–36 porins; modification of the lipopolysaccharide lipid A moiety, through *pmrCAB* mutations or *lpxACD* inactivation, underlies colistin resistance [9,10]. Because OXA-mediated hydrolysis is efficient against both imipenem and meropenem, resistance to the two agents typically moves in parallel. In *P. aeruginosa*, by contrast, carbapenem resistance is more often mutational than acquired. Loss or down-regulation of the substrate-specific OprD porin raises imipenem MICs disproportionately. Meropenem resistance depends more on overexpression of the MexAB-OprM efflux system, on derepression of the chromosomal AmpC cephalosporinase, and, where present, on acquired metallo-β-lactamases such as VIM, IMP and NDM [11,12]. Imipenem and meropenem resistance can therefore diverge in this species. *S. maltophilia* carries chromosomally encoded L1 metallo-β-lactamase and L2 serine β-lactamase, which together confer intrinsic resistance to virtually all β-lactams including carbapenems. It also carries the SmQnr pentapeptide-repeat protein and multiple efflux pumps [13].

The World Health Organization Bacterial Priority Pathogens List, updated in 2024, classifies carbapenem-resistant *A. baumannii* in the critical priority group. Carbapenem-resistant *P. aeruginosa* was removed from the critical group of the 2017 list and placed in the high-priority group in this update [14]. Reported resistance rates in both organisms have continued to rise, a trend attributed in part to the broad-spectrum antimicrobial use that accompanied the COVID-19 pandemic [2,14,15].

Much of the published surveillance from Turkey predates this period [16,17]. This study characterises NFGNB distribution and carbapenem resistance in the adult ICUs of a foundation university hospital in Istanbul between 2023 and 2025. Year-specific EUCAST breakpoint versions were applied, susceptibility categories are reported separately, and the analysis accounts explicitly for repeated isolation from the same patient.

## 2. Materials and Methods

### 2.1. Setting, Design and Population

This retrospective observational study was conducted at a foundation university hospital in Istanbul. All respiratory specimens submitted to the clinical microbiology laboratory from adult ICUs between 6 January 2023 and 27 December 2025 were eligible. Adult status was defined as age ≥ 18 years at the time of specimen collection; neonatal and paediatric ICU specimens, and specimens from patients under 18 years recorded in adult units, were excluded. Data were retrieved exclusively from the laboratory information system and the hospital automation system; individual medical records were not reviewed, and no clinical, treatment or outcome data were available.

**Figure 1:**
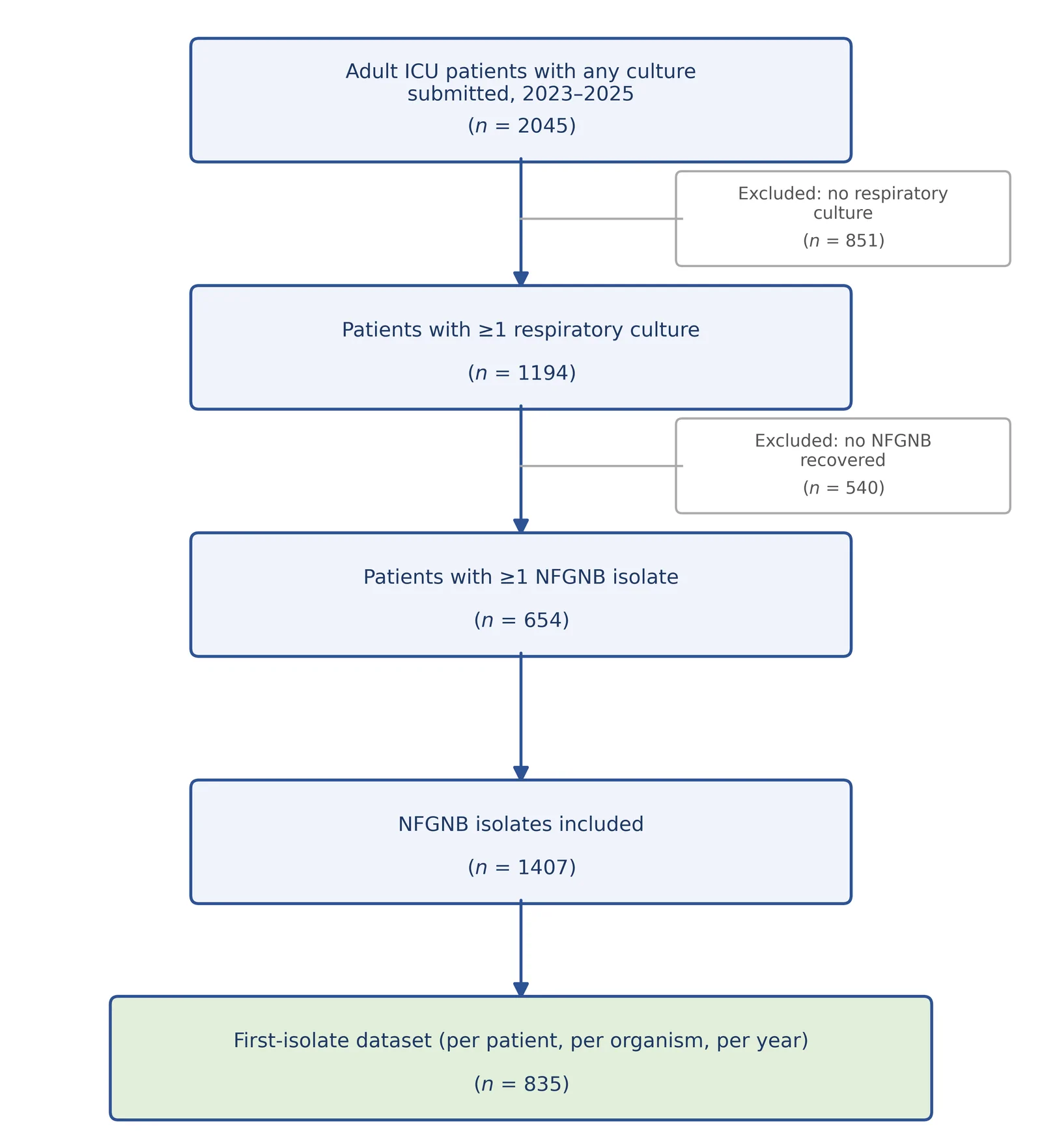
Flow of adult ICU patients and NFGNB isolates through the study. ICU, intensive care unit; NFGNB, non-fermenting Gram-negative bacilli.

Respiratory specimens comprised endotracheal/tracheal aspirates, sputum and bronchoalveolar lavage (BAL). Two test codes exist in the laboratory information system for aspirate specimens, ‘quantitative culture of respiratory secretions’ and ‘tracheal aspirate culture’, and both refer to the same specimen type. The former is the pre-existing default code and was used for the large majority of requests, so aspirate specimens were analysed as a single category. The flow of patients and isolates through the study is shown in Figure 1.

### 2.2. Identification and Antimicrobial Susceptibility Testing

Identification and antimicrobial susceptibility testing were performed with the BD Phoenix M50 automated system using NMIC/ID-492 panels (Becton Dickinson, Sparks, MD, USA), with quality control performed using *Pseudomonas aeruginosa* ATCC 27853. Matrix-assisted laser desorption/ionisation time-of-flight mass spectrometry was not available at the study centre; *Acinetobacter* isolates were therefore reported at the level of the *A. baumannii/calcoaceticus* complex and cannot be resolved to species.

Susceptibility results were interpreted according to the EUCAST breakpoint tables in force in each calendar year: v13.0 for 2023, v14.0 for 2024 and v15.0 for 2025 [18,19,20]. Year-wise trend analysis was restricted to imipenem and meropenem. The MIC breakpoints for these agents were identical across v13.0, v14.0 and v15.0: for *Acinetobacter* spp., imipenem S ≤ 2/R > 4 mg/L and meropenem S ≤ 2/R > 8 mg/L; for *P. aeruginosa*, imipenem S ≤ 0.001/R > 4 mg/L and meropenem S ≤ 2/R > 8 mg/L. Year-wise comparison is therefore based on a constant interpretive framework. All other agents are reported as pooled proportions, with each isolate having been interpreted under the version current at the time of testing.

The results are reported in the three EUCAST categories: S (susceptible, standard dosing regimen), I (susceptible, increased exposure) and R (resistant). No combined ‘susceptibility’ percentage is given. For many agents most isolates fall into category I, and either combining S with I or reporting S alone would misrepresent these agents.

Colistin minimum inhibitory concentrations were determined by broth microdilution using the UMIC Colistin system (UM-COL-040; Bruker Daltonics/MERLIN, Bremen, Germany) with Mueller–Hinton broth, in accordance with the manufacturer’s instructions and with ISO 20776-1 [21,22], using the quality control strains recommended by the manufacturer.

Intrinsic resistance and the absence of breakpoints were taken into account. Ertapenem was excluded because it lacks activity against *P. aeruginosa* and *Acinetobacter* spp. Carbapenems and colistin were not evaluated for *S. maltophilia*, which is intrinsically resistant to both. Piperacillin–tazobactam and tigecycline were not evaluated for *Acinetobacter* spp., and no agent other than trimethoprim–sulfamethoxazole was evaluated for *S. maltophilia*: the EUCAST tables assign no breakpoints for these organism–agent combinations, listing them as ‘insufficient evidence’ or omitting them entirely, so no interpretive category can be derived. EUCAST specifies a separate, lower meropenem breakpoint for meningitis (R > 2 mg/L) than for other indications (R > 8 mg/L). Two results had been reported under the meningitis breakpoint and were excluded, since the present analysis concerns respiratory specimens.

### 2.3. Isolate Definition and Denominators

An isolate was defined by the combination of patient, calendar day and organism; specimens of different types yielding the same organism from the same patient on the same day were counted once. Isolates of the same organism recovered from the same patient on different days were retained as separate isolates in the primary dataset. This departs from the first-isolate approach recommended by CLSI M39 for cumulative antibiograms [23]. The objective was to describe temporal resistance patterns across the whole specimen stream rather than to construct a cumulative antibiogram. The consequences of this choice are quantified in the sensitivity analysis and addressed in Limitations.

Denominators differ between agents and are reported individually for each. Two mechanisms account for this. First, under the national restricted-reporting policy, the results for carbapenems, ceftazidime–avibactam, ceftolozane–tazobactam and cefoxitin are released following infectious diseases review. Reporting coverage nevertheless exceeded 95% for every restricted agent analysed here, so this policy did not materially reduce the available data. Second, colistin is used as a last-line agent in the study laboratory. It is therefore tested not in all isolates but only in those in which prior results have shown multidrug or extensive drug resistance. This is a potential source of selection bias, which is addressed in Limitations.

### 2.4. Statistical Analysis

Because 268 of the 654 patients contributed more than one isolate, observations are not independent, and a conventional chi-square test of the pooled dataset would understate the standard errors. The primary analysis of temporal trend therefore used generalised estimating equations (GEE) with a binomial family, logit link and exchangeable working correlation structure, clustering on patient identity, with calendar year entered as a linear term [24]. The results are expressed as the odds ratio (OR) for resistance per additional calendar year with its 95% confidence interval.

Two supporting analyses are reported. The Pearson chi-square tests across the three years are presented as a secondary, unadjusted comparison. A sensitivity analysis repeated the GEE models within a first-isolate dataset comprising the earliest isolate of each organism per patient per calendar year, which conforms to the CLSI M39 principle while preserving year-wise structure. Proportions are presented with Wilson 95% confidence intervals. Analyses were performed in Python 3.12 using statsmodels 0.14 and SciPy 1.17; two-sided *p* < 0.05 was considered significant.

### 2.5. Ethics

This study was conducted in accordance with the Declaration of Helsinki and was approved by the Non-Interventional Scientific Research Ethics Committee of Istanbul Atlas University (approval no. E-22686390-050.99-99415; 11 May 2026). The requirement for informed consent was waived because of the retrospective design and the use of anonymised laboratory data.

During preparation of this manuscript the authors used AI-assisted tools for language editing and for checking the internal consistency of tabulated data. All output was reviewed and edited by the authors, who take full responsibility for the content of this publication.

## 3. Results

### 3.1. Patients and Specimens

During the study period, 2045 adult ICU patients had at least one culture of any type submitted; 1194 of these had at least one respiratory culture and were eligible. NFGNB were recovered from 654 of them (54.8%), yielding 1407 isolates (Figure 1). The median age was 75 years (interquartile range 66–83; range 20–104). Aspirate specimens accounted for 1329 isolates (94.5%), sputum for 64 (4.5%) and BAL for 14 (1.0%). Patient and specimen characteristics are summarised in Table 1.

A total of 268 patients (41.0% of NFGNB-positive patients) had the same organism recovered on more than one day, contributing 890 isolates in total, of which 587 (41.7% of all isolates) were isolations beyond the first for that patient and organism. The corresponding first-isolate dataset comprised 835 isolates.

**Table 1:** Patient and specimen characteristics.

Characteristic	Value
***Patients (n = 654)***	
Age, years—median (middle 50%)	75 (66–83)
Age, years—youngest and oldest	20–104
Patients with more than one isolate of the same organism	268 (41.0%)
***Isolates (n = 1407)***	
Aspirate (respiratory secretion and tracheal aspirate)	1329 (94.5%)
Sputum	64 (4.5%)
Bronchoalveolar lavage	14 (1.0%)
Isolations beyond the first for that patient and organism	587 (41.7%)
First-isolate dataset	835 (59.3%)

Percentages are calculated within the denominator given in each group heading. The first-isolate dataset comprises the earliest isolate of each organism per patient per calendar year.

### 3.2. Distribution of NFGNB

The *Acinetobacter baumannii/calcoaceticus* complex was the most frequently recovered organism, accounting for 801 isolates (56.9%), followed by *Pseudomonas* spp. (413, 29.4%) and *S. maltophilia* (156, 11.1%); together these three groups represented 97.4% of all isolates. Of the 413 *Pseudomonas* isolates, 408 (98.8%) were *P. aeruginosa*, and susceptibility analyses were therefore restricted to this species. Within the *Acinetobacter* group, 782 of 801 isolates (97.6%) belonged to the *A. baumannii/calcoaceticus* complex; the remaining 19 comprised *Acinetobacter* spp. not further identified (*n* = 14), *A. lwoffii* (*n* = 4) and *A. haemolyticus* (*n* = 1). The full distribution is shown in Table 2.

**Figure 2:**
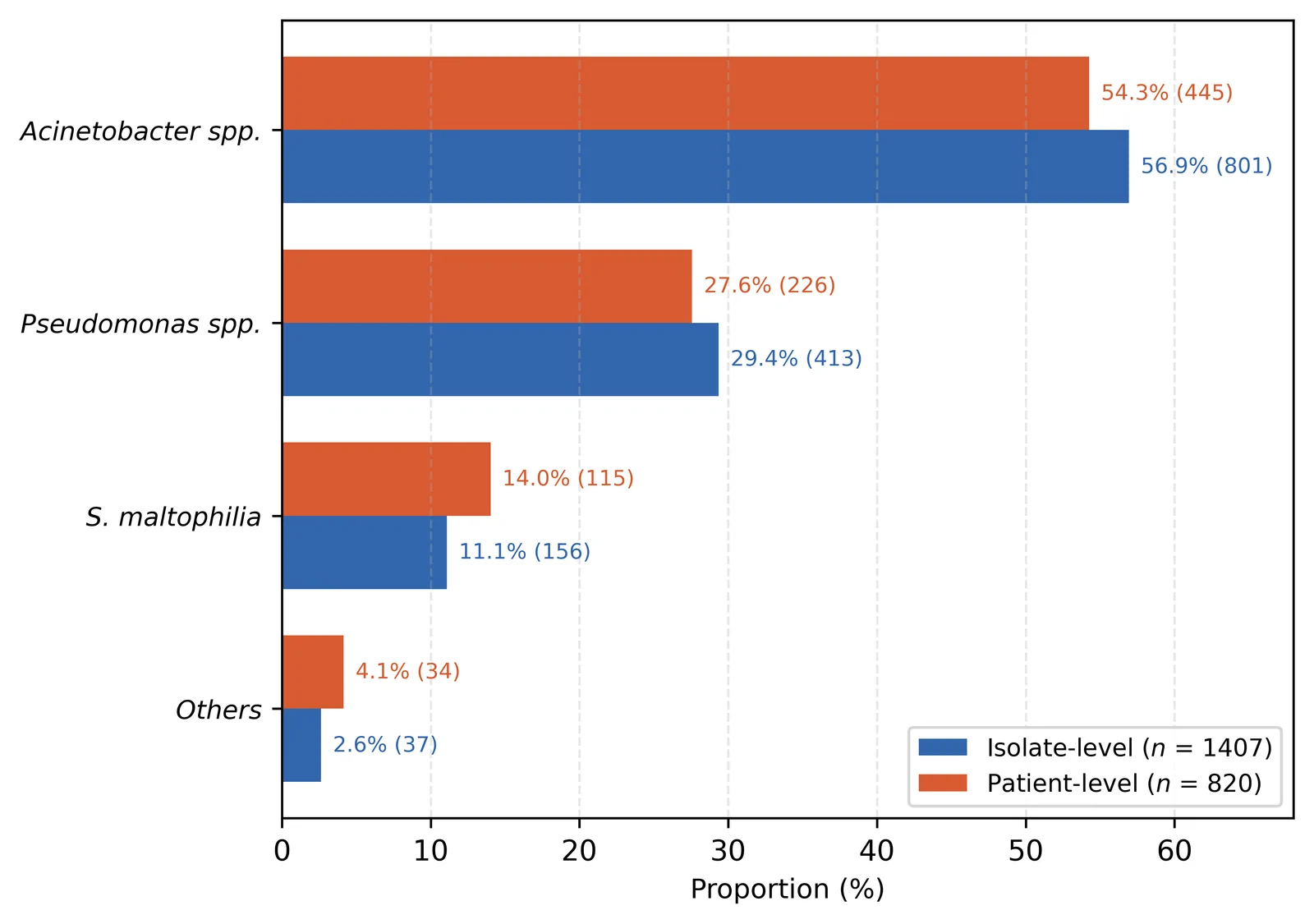
Distribution of the three principal NFGNB groups expressed at isolate level and at patient level.

Because repeated isolation could distort the apparent species distribution, proportions were also calculated at patient level using the first isolate of each organism per patient. The two distributions were closely similar (*Acinetobacter* spp. 56.9% of isolates versus 54.3% of patients; *Pseudomonas* spp. 29.4% versus 27.6%; *S. maltophilia* 11.1% versus 14.0%), indicating that repeated sampling did not materially alter the ranking of species (Table 2, Figure 2).

### 3.3. Antimicrobial Susceptibility

Susceptibility results are presented in Table 3, with S, I and R categories reported separately. In *Acinetobacter* spp., resistance was 96.7% (95% CI 95.3–97.8) for imipenem, 96.6% (95.1–97.7) for meropenem and 93.9% (92.0–95.3) for amikacin. Colistin was the most active agent tested, with 22.4% resistance (19.4–25.8).

In *P. aeruginosa*, imipenem resistance was 66.8% (62.1–71.3) and meropenem resistance 54.8% (49.9–59.7). Only one isolate was categorised as susceptible to imipenem at standard dosing, whereas 132 (32.9%) fell into category I. The same pattern applies to ceftazidime (six susceptible, 154 category I) and piperacillin–tazobactam (four susceptible, 168 category I), for which resistance was 60.1% and 56.8% respectively. This reflects the EUCAST breakpoint structure rather than the isolates: for all three agents the susceptible breakpoint against *P. aeruginosa* is set at the arbitrary off-scale value of ≤0.001 mg/L, which places wild-type organisms in category I by design. The two categories must therefore be read together for these agents. Ceftazidime–avibactam was the most active β-lactam, with 24.3% resistance (20.3–28.8), and colistin was the most active agent overall (13.1%, 9.7–17.5).

For *S. maltophilia*, 133 of 155 isolates (85.8%) tested against trimethoprim–sulfamethoxazole were categorised as I, with only 8 (5.2%) as S and 14 (9.0%) as R. The distribution was stable across the three years (category I: 91.2%, 80.3% and 88.9% in 2023, 2024 and 2025), indicating that pooling did not obscure a temporal shift. Trimethoprim–sulfamethoxazole is the only agent for which EUCAST provides breakpoints against this species, so no other agent could be reported.

**Table 3:** Antimicrobial susceptibility of the principal NFGNB, reported in separate EUCAST categories.

Antibiotic	Tested (*n*)	S	I	R	Resistant % (95% CI)
***Acinetobacter*** **spp. (*****n*** **= 801)**					
Imipenem	799	23	3	773	96.7 (95.3–97.8)
Meropenem	795	25	2	768	96.6 (95.1–97.7)
Amikacin	797	48	1	748	93.9 (92.0–95.3)
Colistin †	646	501	0	145	22.4 (19.4–25.8)
***Pseudomonas aeruginosa*** **(** ***n*** **= 408)**					
Imipenem	401	1	132	268	66.8 (62.1–71.3)
Meropenem	392	142	35	215	54.8 (49.9–59.7)
Ceftazidime	401	6	154	241	60.1 (55.2–64.8)
Ceftazidime–avibactam	391	296	0	95	24.3 (20.3–28.8)
Piperacillin–tazobactam	398	4	168	226	56.8 (51.9–61.6)
Amikacin	396	273	0	123	31.1 (26.7–35.8)
Colistin †	290	250	2	38	13.1 (9.7–17.5)
***Stenotrophomonas maltophilia*** **(** ***n*** **= 156)**					
TMP-SMX	155	8	133	14	9.0 (5.5–14.6)

S, susceptible (standard dosing regimen); I, susceptible (increased exposure); R, resistant; CI, confidence interval. Denominators differ between agents; see Section 2. o. † In the study laboratory colistin is tested as a reserve agent only where prior results indicate multidrug or extensive drug resistance; see Limitations.

### 3.4. Temporal Trends

**Figure 3:**
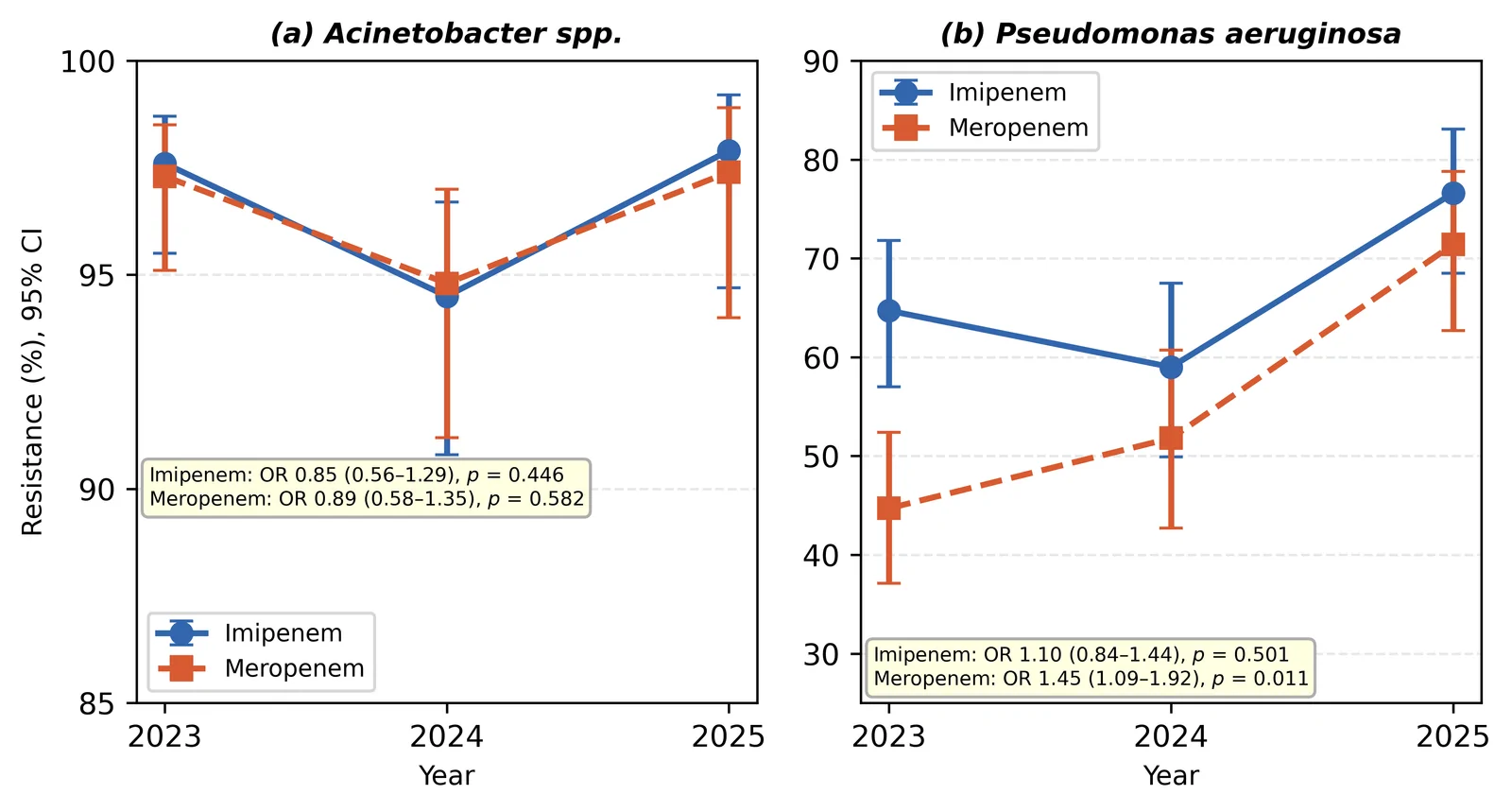
Annual carbapenem resistance with 95% confidence intervals in (**a**) *Acinetobacter* spp. and (**b**) *Pseudomonas aeruginosa*. Odds ratios are from generalised estimating equations accounting for within-patient clustering.

Annual resistance proportions with 95% confidence intervals and the corresponding GEE estimates are shown in Table 4 and Figure 3. In *Acinetobacter* spp., resistance remained above 94% in every year for both carbapenems, and there was no temporal trend after accounting for within-patient clustering (imipenem OR per year 0.85, 95% CI 0.56–1.29, *p* = 0.446; meropenem OR 0.89, 0.58–1.35, *p* = 0.582). The unadjusted chi-square tests were likewise non-significant for meropenem (*p* = 0.213) and borderline for imipenem (*p* = 0.063).

In *P. aeruginosa*, meropenem resistance increased from 44.7% (37.1–52.4) in 2023 to 51.8% (42.7–60.7) in 2024 and 71.4% (62.7–78.8) in 2025. This trend remained significant after adjustment for clustering (OR per year 1.45, 95% CI 1.09–1.92, *p* = 0.011). Imipenem resistance showed no consistent direction (64.7%, 59.0% and 76.6%). The unadjusted chi-square test was significant (*p* = 0.011), but the GEE estimate was not (OR 1.10, 0.84–1.44, *p* = 0.501). The apparent difference is therefore attributable to repeated isolation rather than to a genuine temporal change.

In the first-isolate sensitivity analysis, the direction and magnitude of the *P. aeruginosa* meropenem trend were preserved (36.7%, 44.3% and 51.0%; OR per year 1.37, 95% CI 0.97–1.92, *p* = 0.070). Statistical significance was lost as the dataset contracted from 392 to 226 observations. None of the other three trends approached significance in either analysis. Taken together, the results indicate a real but modest increase in meropenem resistance in *P. aeruginosa*, part of whose apparent magnitude in the unadjusted analysis derives from repeated sampling of the same patients.

**Table 4:** Annual carbapenem resistance and temporal trend estimates.

Organism/Agent	2023% (95% CI)	2024% (95% CI)	2025% (95% CI)	GEE OR per Year (95% CI)	*p*	First-Isolate OR (95% CI), *p*
*Acinetobacter* spp.—imipenem	97.6 (95.5–98.7) *n* = 375	94.5 (90.8–96.7) *n* = 235	97.9 (94.7–99.2) *n* = 189	0.85 (0.56–1.29)	0.446	0.85 (0.54–1.34), *p* = 0.482
*Acinetobacter* spp.—meropenem	97.3 (95.1–98.5) *n* = 373	94.8 (91.2–97.0) *n* = 233	97.4 (94.0–98.9) *n* = 189	0.89 (0.58–1.35)	0.582	0.84 (0.52–1.35), *p* = 0.464
*P. aeruginosa*—imipenem	64.7 (57.0–71.8) *n* = 156	59.0 (49.9–67.5) *n* = 117	76.6 (68.5–83.1) *n* = 128	1.10 (0.84–1.44)	0.501	0.96 (0.69–1.35), *p* = 0.833
*P. aeruginosa*—meropenem	44.7 (37.1–52.4) *n* = 159	51.8 (42.7–60.7) *n* = 114	71.4 (62.7–78.8) *n* = 119	1.45 (1.09–1.92)	0.011	1.37 (0.97–1.92), *p* = 0.070

GEE, generalised estimating equations with exchangeable working correlation clustering on patient; OR, odds ratio for resistance per additional calendar year. The unadjusted chi-square results across the three years were: *Acinetobacter* spp. imipenem χ^2^ = 5.52, *p* = 0.063; meropenem χ^2^ = 3.09, *p* = 0.213; *P. aeruginosa* imipenem χ^2^ = 9.03, *p* = 0.011; meropenem χ^2^ = 20.32, *p* < 0.001. The first-isolate column reports the same GEE model fitted within the first-isolate dataset.

### 3.5. Sampling Intensity

Annual denominators are given in Table 5. The number of adult ICU patients with a respiratory culture fell from 528 in 2023 to 456 in 2024 and 246 in 2025. This reduction coincided with the institution’s transition to university hospital status on 2 December 2024. Contracting with the national social security institution for the new legal entity was completed on 13 March 2025, and admission of insured patients resumed the following day; monthly admissions returned to their previous level from April 2025 onwards. The number of cultures per patient did not change materially over this period, indicating that the reduction reflects fewer patients rather than incomplete data capture. The proportion of cultured patients from whom NFGNB were recovered remained stable (57.6%, 50.7% and 53.3%), suggesting that the case mix submitted for culture did not shift substantially.

## 4. Discussion

ICU patients are exposed to sustained antimicrobial pressure, prolonged instrumentation and frequent contact with healthcare personnel, and infection prevalence in this population has remained high across successive international surveys [25]. Against this background, NFGNB have become established ICU pathogens, having long been regarded as organisms of limited clinical importance. The shift is documented in five-year trend analyses and in tertiary-centre series from several regions [26,27,28].

In this three-year analysis of adult ICU respiratory specimens, the *A. baumannii/calcoaceticus* complex was the organism most frequently recovered, and carbapenem resistance within it exceeded 96% and did not change over time. *P. aeruginosa* showed a different pattern: imipenem resistance was stable, whereas meropenem resistance rose by roughly 45% in relative odds per year. Colistin remained the most active agent against both organisms, and ceftazidime–avibactam retained activity against three-quarters of *P. aeruginosa* isolates.

The predominance of *Acinetobacter* spp. is consistent with reports from Turkey and from other high-prevalence settings. A national point-prevalence study identified *Acinetobacter* spp. as the leading pathogen in ICU infections [29], and a recent Turkish series reported *A. baumannii* as the most frequent isolate in post-pandemic ICU respiratory cultures [15]. A nine-year Italian review similarly found *A. baumannii* to predominate in ICU respiratory specimens [30]. Earlier series, by contrast, described *P. aeruginosa* and *Staphylococcus aureus* as dominant with *Acinetobacter* spp. in a minor role [4,31,32], and studies from Japan, Italy and Bahrain report distributions in which *P. aeruginosa* or *Klebsiella* spp. predominate [33,34,35]. Local surveillance therefore remains indispensable, since the species distribution cannot be assumed from published data.

Data from the Americas describe comparable pressures. Carbapenem-resistant *A. baumannii* increased in tracheal aspirates in Brazil during the pandemic period [36], Latin American surveillance identifies *P. aeruginosa* and *A. baumannii* as leading ICU pathogens [37], and Brazilian national data document a sustained rise in plasmid-mediated carbapenem resistance across Gram-negative species [38].

The differences observed between the two major microorganisms are consistent with differences in their underlying resistance mechanisms. In the *A. baumannii/calcoaceticus* complex, OXA-type carbapenemases hydrolyse imipenem and meropenem with similar efficiency, and resistance to the two agents therefore tended to occur in parallel, remaining close to saturation throughout the study period [9,10]. Resistance rates exceeding 90% have been reported from Italy and Iran [30,39], while comparative analyses consistently identify both organisms among the multidrug-resistant pathogens of greatest concern in intensive care units [40]. Our findings fall at the upper end of this reported range.

In *P. aeruginosa*, however, resistance to imipenem and meropenem is mediated, at least in part, by different mechanisms. Loss of OprD has a disproportionate effect on imipenem susceptibility, whereas overexpression of MexAB-OprM, AmpC derepression, and acquired metallo-β-lactamases may contribute more substantially to meropenem resistance [11,12]. The pattern observed in our study, a relatively stable level of imipenem resistance accompanied by an increasing trend in meropenem resistance, is therefore compatible with a growing contribution of efflux- or enzyme-mediated resistance mechanisms. This remains an inference: no molecular characterisation was performed in the present study, and phenotypic data alone cannot establish the specific mechanisms responsible for the observed resistance patterns.

The magnitude of the observed meropenem trend was strongly influenced by how repeated isolates were handled. In the unadjusted analysis, the increase appeared pronounced and highly significant. After accounting for within-patient clustering, the effect remained but was attenuated. In the first-isolate dataset, the direction of the trend was preserved, but statistical significance was lost because of the smaller sample size.

These findings suggest that the isolate-based analysis captured a genuine signal of increasing meropenem resistance, although its apparent magnitude was likely amplified by repeated sampling of patients carrying resistant strains. The same principle may explain why the imipenem trend in *P. aeruginosa*, which was statistically significant in the unadjusted analysis, was no longer significant after adjustment for within-patient clustering. This pattern highlights an important limitation of surveillance analyses that pool all isolates without accounting for clustering: temporal changes in resistance may be overestimated when patients with repeated isolation of resistant organisms are disproportionately represented [23].

Whether repeated isolates obtained from the same patient represent persistence of a single clone or acquisition of a distinct strain cannot be determined on the basis of phenotypic data alone. Demonstrating clonal relatedness would require molecular typing methods such as pulsed-field gel electrophoresis, multilocus sequence typing, or whole-genome sequencing. Both scenarios are plausible in the context of our findings: resistance may have emerged under antimicrobial selection pressure between successive isolates, or repeated sampling may simply have captured an already resistant strain.

Colistin retained the greatest in vitro activity against both principal organisms, with resistance of 22.4% in *Acinetobacter* spp. and 13.1% in *P. aeruginosa*. Comparable and generally lower figures have been reported from Italy [30] and Turkey [15], and our rates should be read in the light of the selective testing strategy described below. Ceftazidime–avibactam was the most active β-lactam agent against *P. aeruginosa*, with 75.7% of isolates non-resistant. Because this agent has no activity against OXA-producing *A. baumannii*, its relevance here is confined to *P. aeruginosa*. Cefiderocol, sulbactam–durlobactam and tigecycline were not evaluated in this analysis. Sulbactam–durlobactam and tigecycline are options for *A. baumannii* but not for *P. aeruginosa*, which is intrinsically resistant to tigecycline. Cefiderocol has activity against both [9,41]. Newer agents should therefore be considered organism by organism rather than as a single therapeutic category for NFGNB as a whole.

The *S. maltophilia* findings further illustrate why EUCAST susceptibility categories should be reported separately. Only 5.2% of isolates were classified as susceptible to trimethoprim–sulfamethoxazole at standard dosing. Considered in isolation, this figure could give the misleading impression of near-universal resistance. In contrast, 85.8% of isolates were classified in category I, indicating that clinical efficacy is expected when the agent is administered at an increased dosing regimen. Reporting a single ‘susceptibility’ percentage for *S. maltophilia* would therefore have provided an incomplete picture of the organism’s therapeutic options.

Trimethoprim–sulfamethoxazole is currently the only antimicrobial agent for which EUCAST provides specific clinical breakpoints for *S. maltophilia*, further highlighting the limitations in the available framework for assessing antimicrobial susceptibility in this species. The intrinsic resistance of *S. maltophilia* to carbapenems, primarily mediated by the chromosomally encoded L1 metallo-β-lactamase and L2 serine β-lactamase, explains why these agents were excluded from the analysis for this species [13].

A single isolate of *Elizabethkingia meningoseptica* was recovered. No organism-specific conclusion can be drawn from one isolate. The species is nonetheless an emerging nosocomial pathogen with intrinsic carbapenem resistance and a heterogeneous resistance profile and warrants monitoring [42].

The reduction in respiratory cultures over the study period, from 528 patients in 2023 to 246 in 2025, has an identifiable administrative cause. The hospital acquired university hospital status on 2 December 2024. Contracting with the national social security institution for the new legal entity was completed on 13 March 2025, and admissions of insured patients resumed the following day. ICU admissions were correspondingly lower between December 2024 and March 2025 and returned to their previous level from April 2025. Because the number of cultures per patient remained stable and the proportion of cultured patients yielding NFGNB did not change materially, the reduction appears to reflect fewer admissions rather than incomplete data capture or a shift in culturing practice. Nevertheless, the smaller denominators in 2025 reduce the precision of that year’s estimates, as the confidence intervals in Table 4 show.

### Limitations

This is a single-centre retrospective study. The findings describe one institution rather than national or regional epidemiology. Culture positivity in an aspirate or sputum specimen does not by itself establish invasive infection. We had no clinical criteria, radiological findings or treatment data, so we could not separate colonisation from infection. The proportions reported here describe organisms recovered from the respiratory tract of ICU patients; they are not the resistance profile of clinically confirmed infections. Clinical outcomes such as mortality, length of ICU stay and treatment response were not present in the data obtained from the hospital automation system, so the clinical impact of the observed resistance could not be assessed directly.

The isolate-based design counts specimens obtained from the same patient on different days separately. This departs from the first-isolate approach recommended in CLSI M39 for cumulative antibiograms [23]. We addressed this by modelling patient clustering explicitly in the primary analysis and by reporting a first-isolate sensitivity analysis; readers wishing to compare our figures with published cumulative antibiograms should use the first-isolate results in Table 4. Because clonal relatedness was not assessed, it could not be determined how much of the difference reflects repeated sampling of a single strain and how much reflects resistance acquired over time.

Three limitations come from how the laboratory works. Colistin is a last-line agent, so it is not tested in every isolate, and this creates a selection bias. The data show which way that bias runs: imipenem resistance was 97.7% among the *Acinetobacter* isolates tested for colistin but 91.9% among those not tested, and for *P. aeruginosa* the figures were 70.5% and 61.0%. True colistin resistance across all isolates is therefore probably lower than we report. Resistance was assessed phenotypically. We did not look for carbapenemase genes or efflux-pump expression, so the mechanisms discussed above are inferences from the literature rather than findings of this study. Finally, the exported dataset held categorical results only, which left us unable to examine MIC distributions.

Tigecycline is tested against *Acinetobacter* spp. in the study laboratory. However, it is not reported in this manuscript. EUCAST does not assign MIC or zone diameter breakpoints for this organism–agent combination. The laboratory accordingly reports the measured zone diameter for the clinician’s guidance rather than a susceptibility category. These measurements were not included in the dataset exported for this study, so no interpretable susceptibility data for tigecycline could be derived from it. Finally, the dataset made available for this analysis did not include a unique patient identifier, so patients were matched by name.

## 5. Conclusions

Carbapenem resistance was high among the NFGNB isolated from adult ICU respiratory specimens at this centre. In the *A. baumannii/calcoaceticus* complex it stayed close to 97% across all three years, with no measurable trend, and colistin was the only agent we report here that retained appreciable in vitro activity. Meropenem resistance in *P. aeruginosa* rose over the same period. The increase held after we accounted for repeated isolation from the same patients, though part of its apparent size comes from that repetition. Imipenem resistance did not change. Ceftazidime–avibactam remained active against three-quarters of *P. aeruginosa* isolates, making it the most useful β-lactam option in this collection.

These data describe organisms recovered from respiratory specimens and cannot distinguish colonisation from infection or be linked to clinical outcomes. They should guide institutional empirical-treatment policy and antimicrobial stewardship, always alongside clinical assessment, and should not be taken as evidence of treatment failure.

## Figures and Tables

**Table 2:** Distribution of NFGNB at isolate and patient level.

Organism	Isolates (*n* = 1407)	%	Patients (*n* = 820)	%
*Acinetobacter* spp.	801	56.9	445	54.3
*Pseudomonas* spp.	413	29.4	226	27.6
*Stenotrophomonas maltophilia*	156	11.1	115	14.0
*Burkholderia cepacia complex*	14	1.0	13	1.6
*Achromobacter* spp.	7	0.5	6	0.7
*Sphingomonas paucimobilis*	8	0.6	8	1.0
*Chryseobacterium* spp.	7	0.5	6	0.7
*Elizabethkingia meningoseptica*	1	0.1	1	0.1

Patient-level counts use the earliest isolate of each organism per patient over the whole study period; a patient with two different organisms contributes to both rows.

**Table 5:** Annual sampling intensity in adult ICUs.

Year	Patients with Any Culture	Patients with a Respiratory Culture †	NFGNB-Positive Patients	NFGNB Isolates	NFGNB Positivity (%)
2023	931	528	304	617	57.6
2024	756	456	231	439	50.7
2025	422	246	131	351	53.3

Patients contributing cultures in more than one calendar year are counted in each year. † Respiratory cultures comprise aspirate (respiratory secretion and tracheal aspirate), sputum and bronchoalveolar lavage specimens. Admission counts were not available for this analysis.

## Data Availability

The data presented in this study are available on request from the corresponding author. The data are not publicly available owing to privacy restrictions.

## Abbreviations

The following abbreviations are used in this manuscript:

BAL	Bronchoalveolar lavage
CI	Confidence interval
EUCAST	European Committee on Antimicrobial Susceptibility Testing
GEE	Generalised estimating equations
ICU	Intensive care unit
MIC	Minimum inhibitory concentration
NFGNB	Non-fermenting Gram-negative bacilli
OR	Odds ratio
TMP-SMX	Trimethoprim–sulfamethoxazole
WHO	World Health Organization
